# Anatomic Outside-In Reconstruction of the Anterior Cruciate Ligament Using Femoral Fixation with Metallic Interference Screw and Surgical Staples (Agrafe) in the Tibia: An Effective Low-Cost Technique

**DOI:** 10.2174/1874325001711011154

**Published:** 2017-10-31

**Authors:** Ariel de Lima Diego, de Vasconcelos, Stemberg Martins, Leite, José Alberto Dias, Pinto, Dilamar Moreira, Teixeira, Rogério Beltrão, de Léo, Álvaro Coelho, Silveira, Leonardo de Lima, Gonçalves, Romeu Krause, Gonçalves, Marcelo Carvalho Krause, de Vasconcelos, Ana Carolina Leite, Filho, Carlos Frederico Dias Costa, de Lima, Lana Lacerda

**Affiliations:** 1 ITORK - Institute of Traumatology and Orthopedics Romeu Krause, Recife - PE, 50070-490, Brazil; 2 Universidade Federal do Ceará, Fortaleza, CE Brazil; 3 Hospital Armindo Moura Moreno, PE Brazil

**Keywords:** Reconstruction, Anterior Cruciate Ligament, Low-cost, Interference Screw, Surgical Staple, Agrafe

## Abstract

**Background:**

An anterior cruciate ligament (ACL) rupture is a frequent injury, with short and long-term consequences if left untreated. With a view to benefitting as many patients as possible and preventing future complications, we created a low-cost ligament reconstruction technique.

**Method:**

The present article describes an anatomic ACL reconstruction technique.

**Results:**

The technique involves single-band reconstruction, using flexors tendon graft, outside-in tunnel perforation, femoral fixation with metal interference screw and surgical staples (Agrafe) in the longitudinal position.

**Conclusion:**

We present a simple, easy-to-reproduce technique that, when executed on patients with good bone quality, primarily in the tibia, is effective and inexpensive, favoring its large scale application.

## 1 INTRODUCTION

Anterior cruciate ligament (ACL) rupture is one of the most common sports-related orthopedic injuries, with an annual incidence of 35 per 100,000 individuals [1].

An ACL rupture can be extremely damaging in young athletes. Even in non-athletes, an untreated ACL injury can evolve into osteoarthritis of the knee in around 50% of cases in approximately 12 years, which is particularly disastrous since most of these patients are young [2, 3]. Because of its high incidence, ACL rupture can be considered a public health problem, access to ligament reconstruction being essential to better serve the general public [4].

Generally, it is important to devise new, primarily inexpensive and effective ACL reconstruction techniques in order to preserve short and long-term knee function [5, 6]. Among the main factors for the high cost of the surgery in question is the choice of graft fixation material [7]. Initial fixation maintains the graft in position during biological incorporation, which takes around 12 weeks [8]. A number of fixation techniques have been used to achieve this [9].

In the femur, fixation can be by compression (ex: interference screws), expansion (resulting from the introduction of one or two pins transverse to the femoral tunnel, bending the graft and fixing it due to pressure against the wall) or suspension (obtained cortically, with anchors or a button) [10]. In the tibia, in addition to fixation by compression and expansion like the femur, cortical anchoring (ex: staples and screws) and hybrid types that use more than one fixation method simultaneously, can also be used [10, 11].

**Fig. (1):**
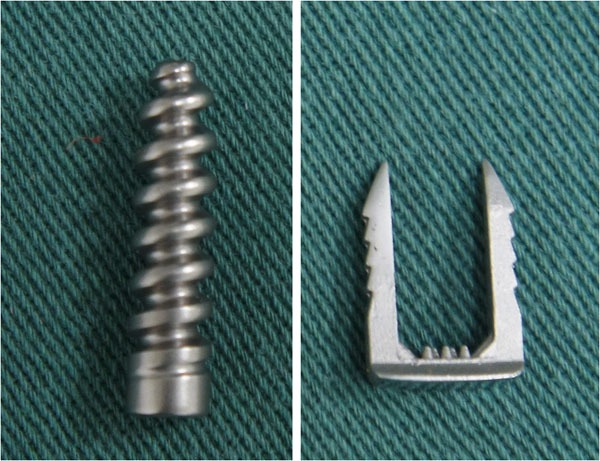
Metallic interference screw and surgical staple (Agrafe).

The aim of the present study is to describe an effective, low-cost ACL reconstruction technique, in which we performed single-band anatomic reconstruction using flexors tendon graft, outside-in tunnel perforation, femoral fixation with metallic interference screw and tibial fixation with surgical staple (Agrafe) in the longitudinal position, anchoring the graft loop (Fig. **1**).

## 2 TECHNIQUE

**Fig. (2):**
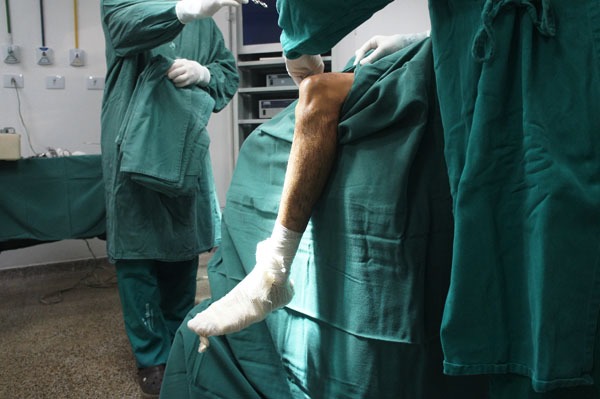
Patient's position.

**Fig. (3):**
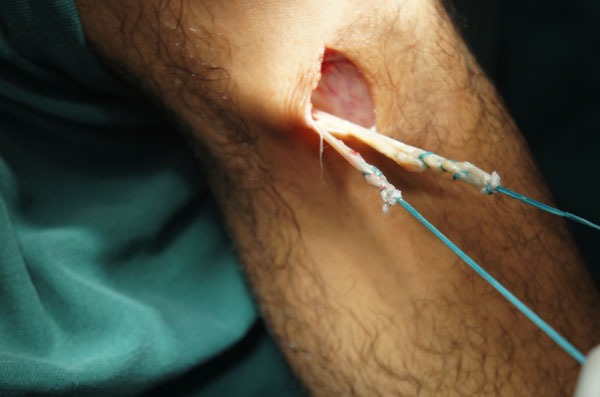
Incision for removal of the flexors graft.

Patients are submitted to regional or general anesthesia and placed in the dorsal decubitus position, with legs hanging over the edge of the operating table and 90° knee flexion (Fig. **2**). An oversleeve is used to produce ischemia, preceded by emptying with the help of an Esmarch band. A 4 cm long oblique incision is made anteromedially to the anterior tibial tuberosity to remove the semitendinosus and gracilis tendons and to construct the tibial tunnel using the outside-in technique (Fig. **3**).

**Fig. (4):**
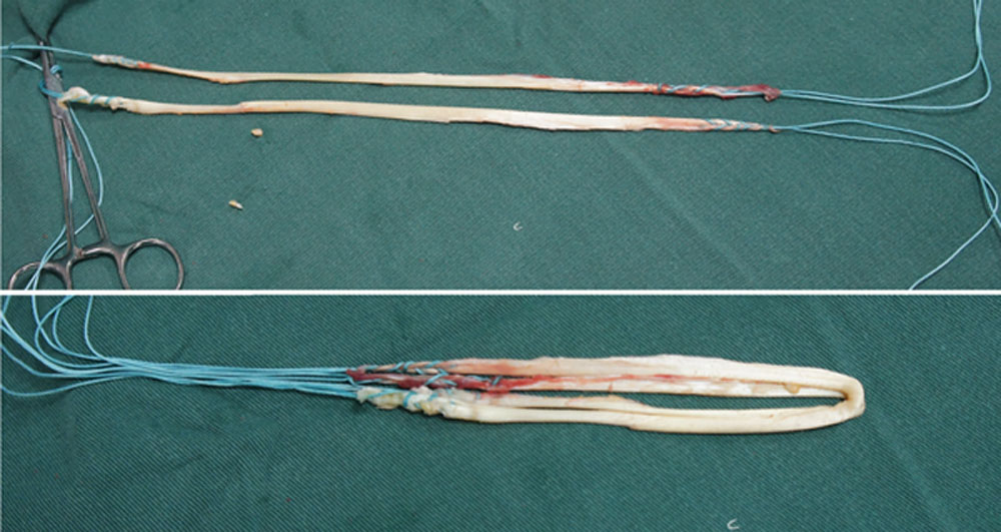
Quadruple flexor tendon graft.

**Fig. (5):**
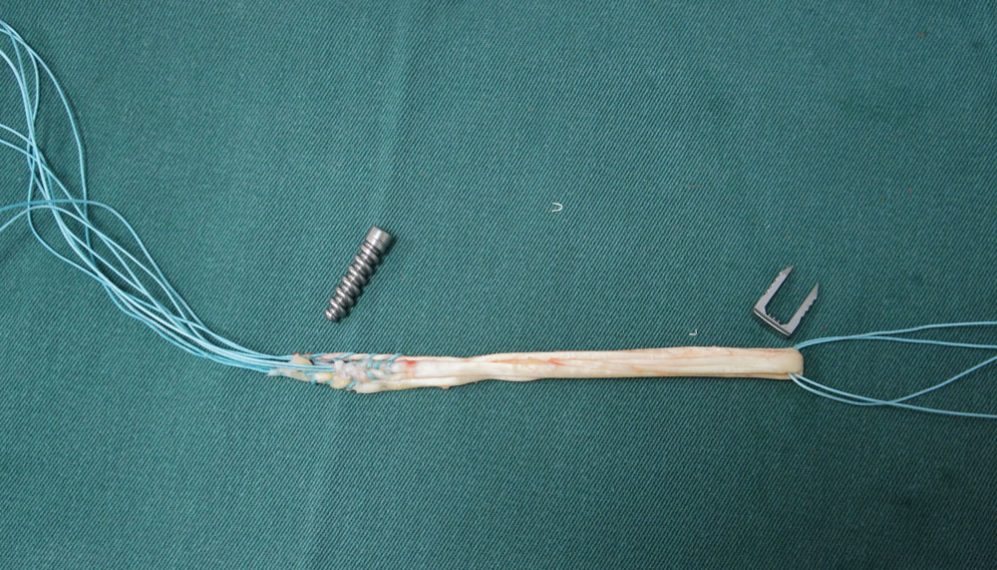
Quadruple flexor tendon graft.

The tendons are harvested with a standard tendon stripper and prepared on an auxiliary table. The ends of the grafts are sutured with a polyester thread. The two tendons are placed side by side and folded in half, so that the four sutured ends are on one side and a loop on the other, forming a quadruple flexor tendon graft (Figs. **4** and **5**).

**Fig. (6):**
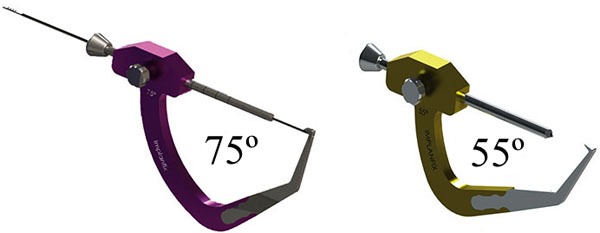
Outside-in guide.

The joint is explored arthroscopically, using an anterolateral portal for the arthroscope and an anteromedial portal for the instruments, and the medial wall of the lateral femoral condyle is prepared using a shaver and curette. Both femoral and tibial tunnels are created in an outside-in manner using a generic guide for anatomic ACL reconstruction. In the femur, we use a guide with angulation of 75º, and in the tibia, a guide of 55º and tunnel creationare preceded by the placement of guidewires (Fig. **6**).

**Fig. (7):**
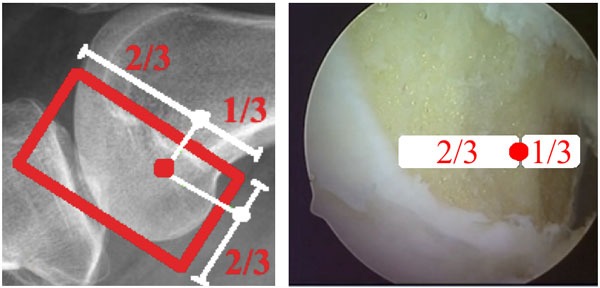
The center of the femoral tunnel.

In the femoral tunnel, the arthroscope is inserted through the anteromedial portal and the guide into the anterolateral portal. Whenever possible, bony landmarks are identified, such as the bifurcate ridge and the resident’s ridge, which helps determine the femoral footprint of the ACL. The center of the tunnel is established in the region of the anteromedial ACL band, posterior to the resident’s ridge, approximately at the point between the anterior 2/3 and posterior 1/3 at the largest diameter of the condylar wall in the sagittal plane and between the superior 1/3 and inferior 2/3 of the height of the condylar wall (Fig. **7**).

**Fig. (8):**
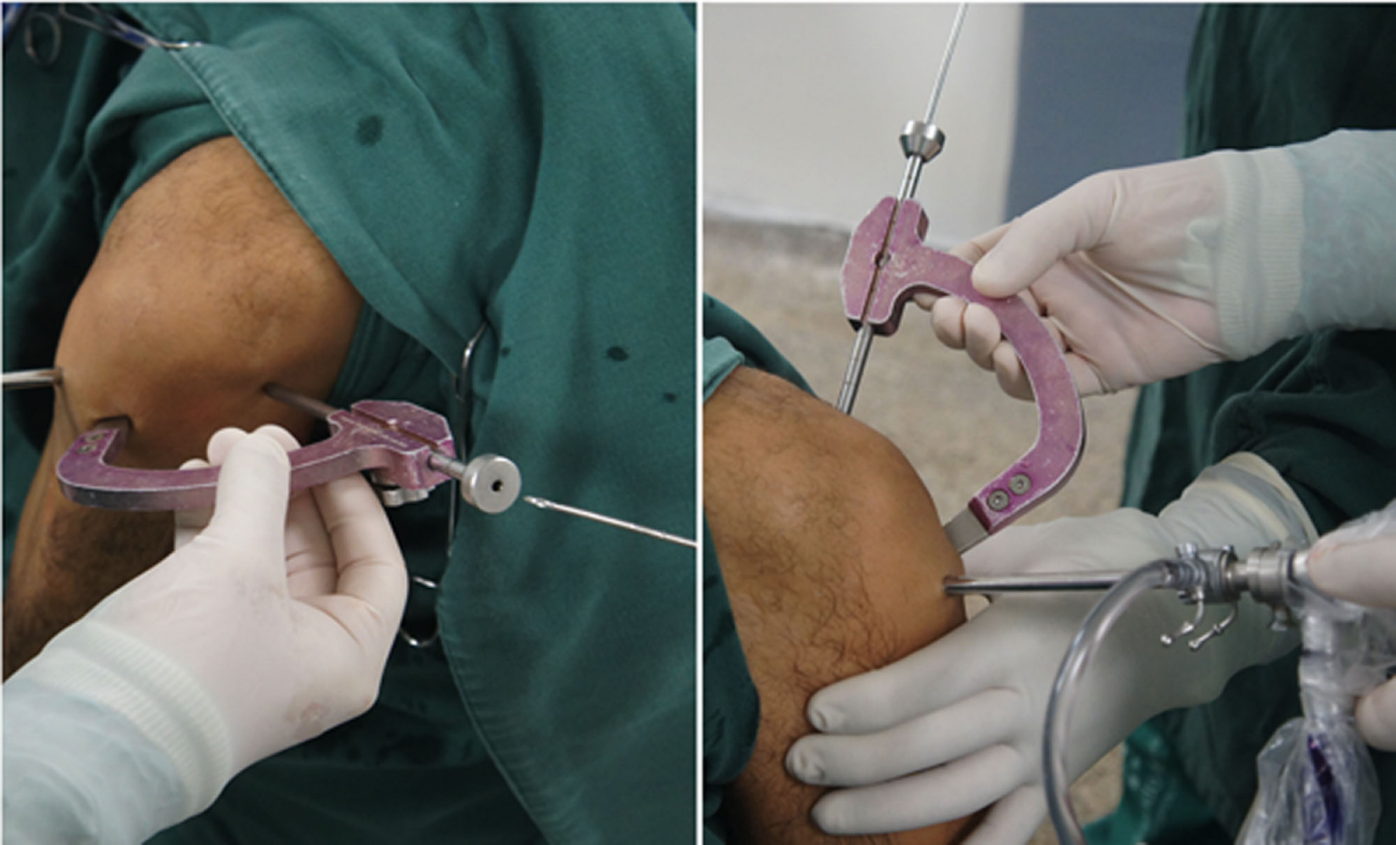
Femoral tunnel.

After identifying the center of the tunnel, the 75º guide, at the anterolateral portal, is inclined superiorly at 60º in the coronal plane and anteriorly at 30º in the sagittal plane. The entry point of the guidewire is marked on the skin and a 2cm incision is made, followed by dieresis up to the cortical bone. With the help of the guide 75º, a guidewire is placed, exiting at a point identified with the intra-articular tip of the femoral guide (Fig. **8**).

**Fig. (9):**
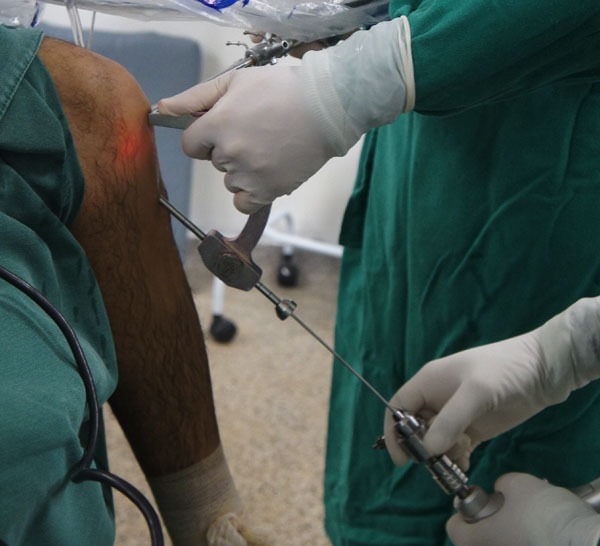
Tibial tunnel.

**Fig. (10):**
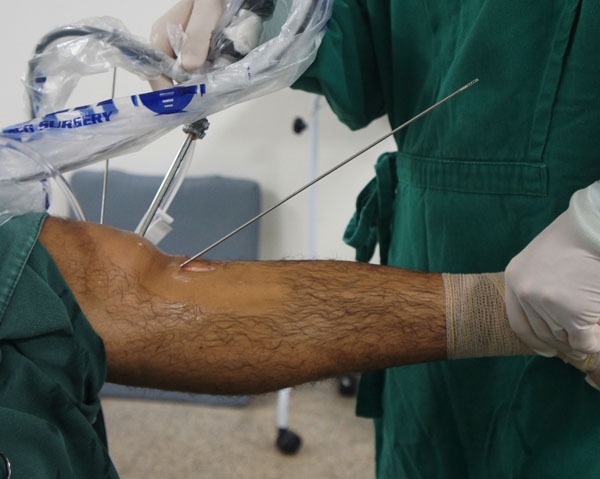
Knee flexion-extension.

In the tibial tunnel, the arthroscope returns to the anterolateral portal and the guide 55º through the anteromedial portal. The guide is placed in the center of the native ACL footprint, medial and posterior to the anterior horn of the lateral meniscus. It is positioned so that the guidewire penetrates the anteromedial cortex of the tibia in the region where the incision was made to remove the flexor tendon grafts, with approximate medial inclination of 30º in the coronal plane (Fig. **9**). After the tibial and femoral guidewires are inserted, knee flexion-extension is performed under arthroscopy in order to identify possible incorrect positioning of the tunnels (Fig. **10**).

Both tunnels are then completed using a drill with the same diameter as the graft.

**Fig. (11):**
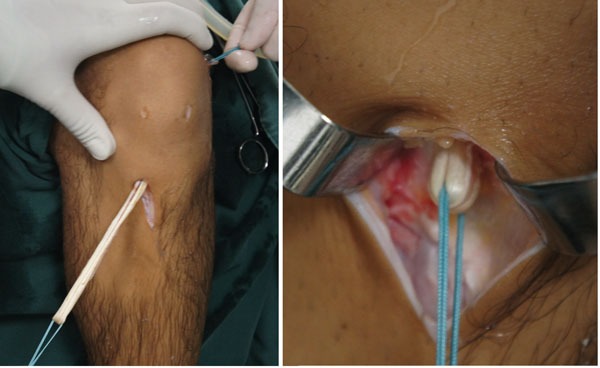
Introduction of the graft.

**Fig. (12):**
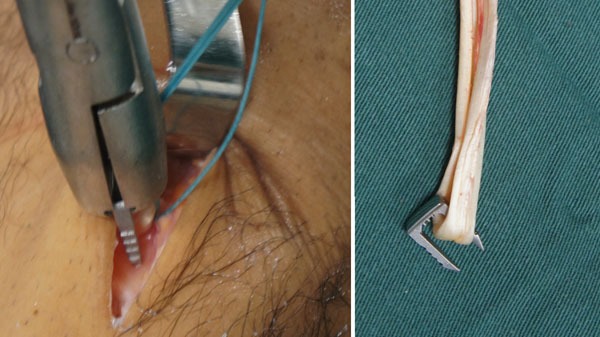
Tibial fixation with Agrafe in the longitudinal position, anchoring the graft loop.

**Fig. (13):**
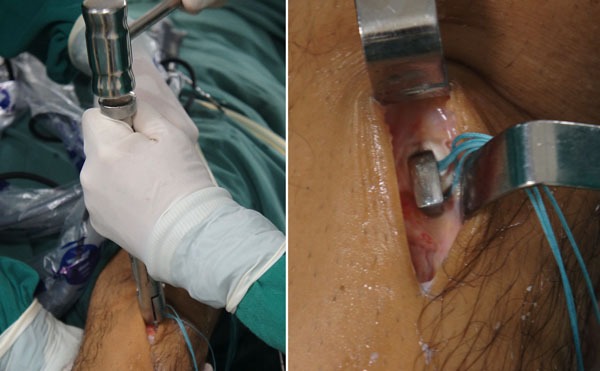
Tibial fixation with Agrafe in the longitudinal position, anchoring the graft loop.

**Fig. (14):**
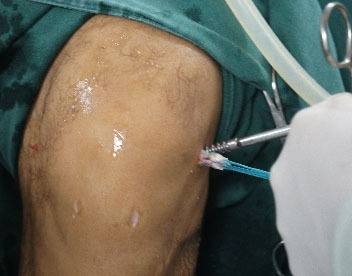
Femoral fixation with metallic interference screw.

The graft is introduced from the caudal to the cranial, such that the loop is in the tibia and the 4 ends sutured with polyester in the femur (Fig. **11**). The loop is fixed to the tibial cortex with a surgical staple (Agrafe), so that one of the “legs” of the staple is inside the loop, functioning as a post (Fig. **12**), with the staple in the longitudinal position, approximately parallel to the tibial tunnel (Fig. **13**). The graft is tensioned through the exit of the femoral tunnel and fixed using metallic interference screw with the same diameter as the tunnel and the maximum length that it allows (Fig. **14**).

**Fig. (15):**
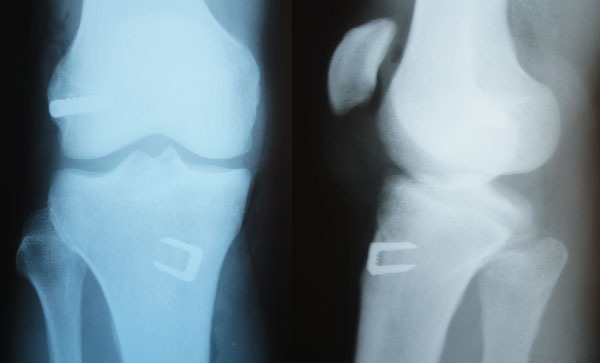
Postoperative radiography.

We retest flexion-extension in order to determine whether there was any blockage or loss of range of motion, and if none is detected, the incision is closed, sterile dressing is applied and no drains are used (Fig. **15**).

## 3 DISCUSSION

Given that ACL rupture is a public health problem, effective low-cost techniques are important to reach a larger number of patients [12].

In 2015, Archibald-Seiffer *et al*. computed the average cost, in USD, of ACL reconstruction [7]. The total cost ranged from USD 392.80 to 4670.31, with an average of USD 2039.09. The cost of tibial fixation varied between USD 95.00 and 760.00 (average of 293.52) and femoral fixation oscillated according to the technique and material used, and is less costly with the use of interference screws than button fixation [7].

With respect to the material used in interference screws, there is no statistically significant difference between clinical results in patients with metallic or bioabsorbable screws. However, the cost of using metallic screws is much lower, making them feasible for use on a large scale [13-15].

**Fig. (16):**
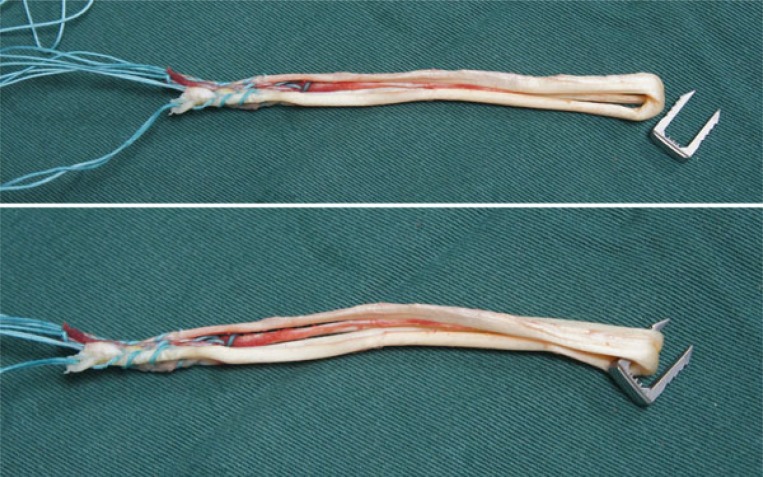
One of the “legs” of the Agrafe is inside the graft loop, functioning as a post.

Tibial fixation with surgical staples (Agrafe) is widely used as an adjuvant to another type of fixation, primarily in patients with open physes. However, in this case staples are used in the transverse position [16, 17]. In the technique we describe, staple is placed longitudinally, using one of the “legs” to anchor the graft loop (Fig. **16**). Thus, we use only one screw in the femur and only one staple in the tibia, reducing the total cost of the implants [7]. However, like the other fixation techniques using cortical anchoring, tibial fixation with surgical staple is indicated for patients with good cortical bone [10, 11]. In relation to the graft, Núñez *et al.* reported that single-band anatomic reconstruction using semitendinosus and gracilis tendons is around 20% cheaper than double-band reconstruction [18].

In regard to the tunnel-drilling technique, Cournapeau *et al*. demonstrated that the all-inside procedure is 17.6% more expensive than the standard technique (tunnel perforated outside to inside in the tibia and the femur) [19].

In 2014, Mall *et al.* conducted an epidemiological study to determine the incidence of ACL reconstruction in the United States [20]. The authors called attention to the growing number of ACL ruptures (especially in women, individuals younger than 20 years of age and adults over the age of 40), underscoring the importance of further research and measures to reduce costs, in order to reach the largest number of patients possible and prevent future complications [20]. We emphasize that this is not an unprecedented technique. There are similar techniques. However, we intend to disclose it because of its importance.

In Brazil, this technique was widely divulged by the authors Pinto and Vasconcelos. Our team has already performed more than 400 ACL reconstructions with this technique. In the future we intend to publish the results in the long term.

## CONCLUSION

Due to the increasing number of ACL injuries, low-cost reconstruction techniques are essential. In the present study we present a simple, easy-to-reproduce technique that is effective and inexpensive when applied to patients with good bone quality, primarily in the tibia, making it feasible for large-scale use.
